# Psychological readiness mediates performance transfer in constraints-led, game-based basketball training: evidence from a quasi-experimental study

**DOI:** 10.3389/fpsyg.2026.1735248

**Published:** 2026-06-11

**Authors:** Chi Ma, Limeng Liu, Yongheng Zhao, Yimeng Gu, Zhongtang Li

**Affiliations:** 1 College of Sports and Health Science, Mudanjiang Normal University, Mudanjiang, China; 2 School of Wushu, Henan University, Kaifeng, China; 3 Graduate School, Kyungil University, Gyeongsan-si, Gyeongbuk, Republic of Korea; 4 School of Physical Education, Jiangsu Second Normal University, Nanjing, China

**Keywords:** basketball athletes, constraints-led training, game-based practice, perception–action coupling, performance transfer, psychological readiness

## Abstract

**Background:**

Constraints-led, game-based training is increasingly used in basketball, yet the behavioral and psychophysiological mechanisms behind performance improvements remain unclear

**Methods:**

A quasi-experimental, pretest–posttest design involved 48 male collegiate basketball players allocated into a constraints-led experimental group (EXP, *n* = 24) or a technique-then-scrimmage control group (CTRL, *n* = 24). Over 8 weeks, both groups trained 3 × /week with matched intensity (70–85% HR_max, sRPE 6–7). External load (distance, PlayerLoad™, accelerations, jumps), internal/perceptual load (HR, fatigue, enjoyment), and technical–tactical performance (FG%, AST/TO) were evaluated using baseline-adjusted ANCOVA, linear mixed-effects models, and bootstrapped mediation/SEM analyses.

**Results:**

EXP showed significantly greater gains in FG% (+ 3.3%, *d* = 0.62) and AST/TO (+ 0.19, *d* = 0.55) versus CTRL (both *p* < 0.01), alongside larger increases in distance (+ 11.4%), PlayerLoad™ (+ 9.6%), and accelerations (+ 13.1%) (all *p* < 0.05). Fatigue decreased while enjoyment increased in EXP, and psychological readiness served as a significant indirect pathway linking load adaptation to performance improvement (indirect effects: β = 0.23–0.41, *p* < 0.05). Multi-group SEM indicated stronger indirect effects among lower-skill players.

**Conclusion:**

A constraints-led model enhances basketball performance through an efficiency-based adaptation profile in which external-load engagement, affective recovery, and improved decision-making are jointly aligned. These results highlight representational fidelity and psychological engagement as synergistic drivers of training responses.

## 1 Introduction

Technical–tactical performance in basketball relies heavily on athletes’ capacity to perceive dynamic environmental cues and translate them into timely, efficient decisions under pressure (Collins and MacNamara, 2012). However, training in many collegiate programs remains dominated by a technique-first approach, where isolated drills precede limited game play. This separation often weakens perception–action coupling, producing skill execution that fails to transfer to competitive situations where spatial–temporal uncertainty is intrinsic.

From an ecological dynamics perspective, skill should not be viewed as a preloaded motor program but as a functional behavior emerging from continuous interactions between the athlete and environment (Pinder et al., 2011; Seifert et al., 2022; Araújo et al., 2025). Game-based, constraints-led models seek to preserve this interaction by manipulating informational variables—such as court spacing, defender proximity, and temporal demand—to promote real-time decision formation (Silva et al., 2013; Kennedy and O’Brien, 2024). Such environments may also enhance motivation and adaptive engagement, critical psychological resources for sustained learning (Headrick et al., 2015).

Existing research has shown positive effects of constraints-led practice on technical and tactical outcomes, yet significant knowledge gaps remain. First, most studies emphasize physical or skill-based outputs while overlooking the psychological processes that may underpin performance transfer. Enjoyment, fatigue regulation, attentional allocation, and perceived challenge are conceivably central to how athletes absorb and apply performance-relevant information (Arcos et al., 2015; Gil-Arias et al., 2019; Ramos et al., 2020; Cao et al., 2021; Gok et al., 2023; Zeng et al., 2023). Second, little is known about whether the effectiveness of perceptual–affective adaptation varies by athletes’ initial competence. Players with less-developed tactical schemas may respond differently to contextual variability than more skilled athletes (Ranganathan and Newell, 2010; Oppici et al., 2017). Third, the literature remains sparse in formal tests of mediation mechanisms, making it unclear whether psychological readiness plays a functional role in performance transfer or merely reflects improved performance (Hayes, 2022).

Therefore, this study examined whether a constraints-led, game-based intervention would induce superior performance transfer compared with traditional technique-then-scrimmage training in collegiate basketball athletes. More specifically, we tested the hypothesis that: (1) the experimental group would exhibit larger improvements in external load engagement and technical–tactical performance; (2) changes in psychological readiness—particularly enjoyment and fatigue—would serve as significant indirect pathways linking load adaptation to performance improvement; and (3) these mediation effects would be more potent in lower-skill athletes, consistent with skill-dependent learning sensitivity.

By integrating objective workload tracking, validated psychological assessments, and structural equation modeling, this study investigates not only whether constraints-led training is practical, but how and for whom it yields performance advantages. These insights aim to support a shift from volume-focused coaching strategies toward adaptive efficiency as a performance development target in basketball.

## 2 Materials and methods

### 2.1 Study design and oversight

**FIGURE 1:**
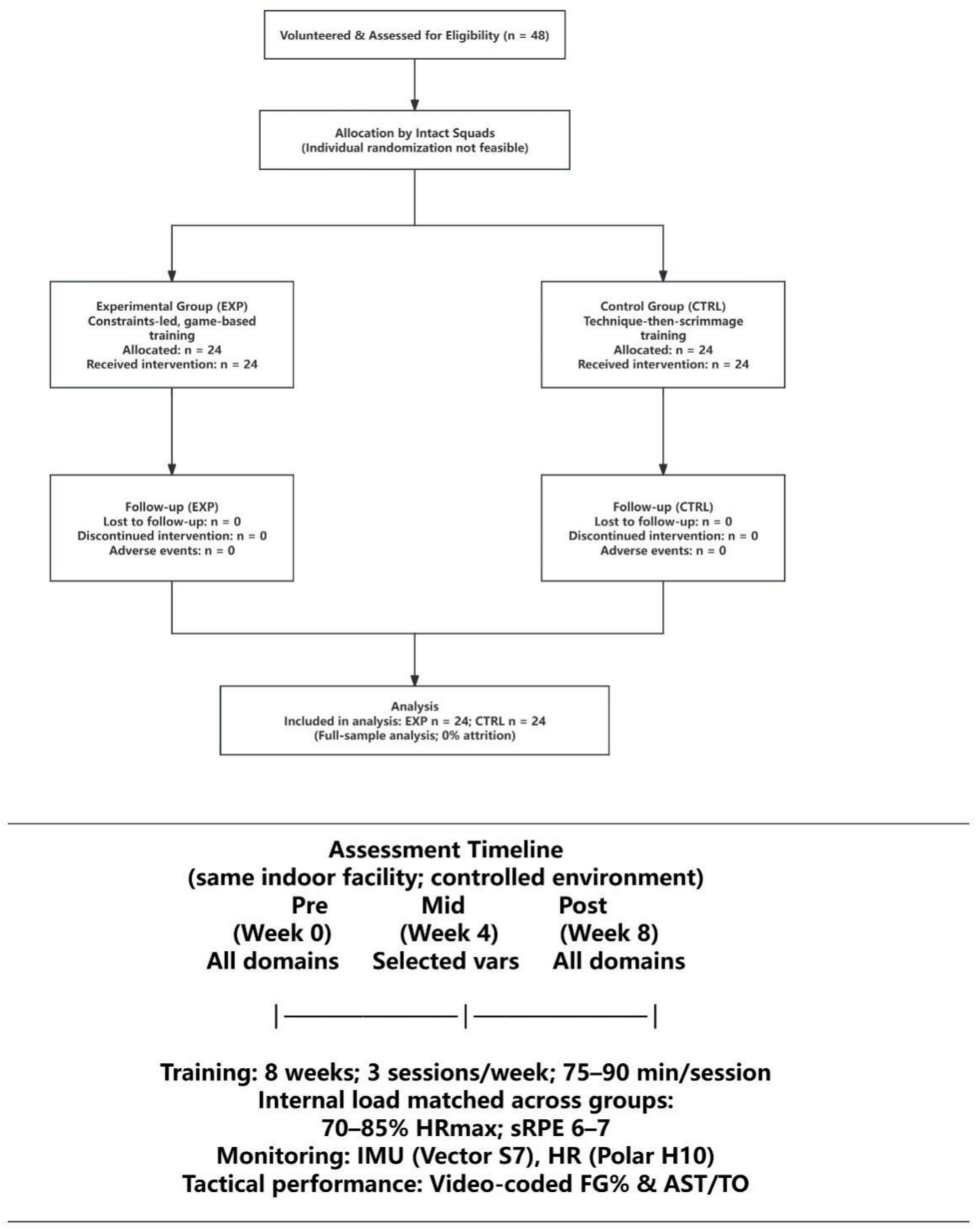
Participant flow and assessment timeline of the intervention. Participants were allocated by intact squads into either the constraints-led, game-based training group (EXP) or the technique-then-scrimmage group (CTRL). All participants completed the 8-week intervention and were included in the full-sample analysis (n = 48). Pre- and post-intervention assessments included all outcome domains, while selected variables were evaluated at mid-intervention (week 4).

We employed a non-equivalent control-group, pretest–posttest quasi-experimental design with group allocation by intact squads, functionally corresponding to a cluster-allocation design at the squad level. Figure 1 provides an overview of participant flow and the Pre/Mid/Post assessment timeline, including screening, allocation by intact squads, intervention exposure, and inclusion in the full-sample analysis (Des Jarlais et al., 2004). Two intact collegiate men’s basketball squads (*n* = 24 each) completed either a constraints-led, game-based intervention (experimental group) or a technique-then-scrimmage program (control group). Individual randomization was not feasible due to fixed squad structures and timetable constraints (Denny et al., 2023).

To reduce selection bias, both squads trained under identical environmental and temporal conditions, using the same equipment, session frequency, and session duration, with no crossover of athletes or coaches. To further reduce contamination and co-intervention risks, the two squads trained in separate time blocks and followed protocol-specific session plans throughout the intervention. Coaches were instructed to deliver only the assigned training model and not to exchange intervention-specific drill structures, constraint rules, or tactical task designs across groups. Athletes were asked not to share structured training content with players from the other squad during the intervention period. Participants were also instructed to avoid additional non-prescribed basketball training, structured skill sessions, or external conditioning programs outside the scheduled team sessions. Weekly self-report checklists were used to monitor additional physical activity, sleep, and recovery-related behaviors. No systematic protocol crossover, additional structured basketball intervention, or moderate-to-severe adverse event was reported during the study period. Baseline comparability was primarily assessed using standardized mean differences, all below 0.20 (trivial imbalance). Welch’s *t*-tests were provided descriptively for continuous variables, and chi-square or Fisher’s exact tests were used for categorical descriptors such as playing position and competitive level when applicable. These analyses supported numerical equivalence between groups (all *p* > 0.05). Primary intervention effects were estimated using baseline-adjusted ANCOVA for Pre–Post outcomes and linear mixed-effects models for repeated data, which statistically controlled for residual differences (Moher et al., 2010). Playing position and competitive level were also included as covariates in sensitivity checks to mitigate further imbalance.

Training analysts blinded to group allocation performed outcome coding for tactical performance. Data were anonymized before statistical analysis to minimize detection bias. Intervention fidelity was monitored using structured weekly checklists, with double ratings of 20% of sessions confirming strong inter-rater reliability (ICC[2,1] = 0.86, 95% CI: 0.79–0.91) (Hoffmann et al., 2014; Lambert et al., 2017).

All training and assessments took place in an indoor university facility in Mudanjiang, China, under controlled environmental conditions (20–23°C; 40–55% relative humidity). The Research Ethics Committee of the School of Physical Education, Mudanjiang Normal University (MDNU-REC-PE-2025-032; 15 April 2025) approved the study protocol. All participants (≥ 18 years) provided written informed consent before enrollment, and the study adhered to the Declaration of Helsinki (2013 revision).

This behavioral training study in healthy adult athletes does not meet the WHO/ICMJE definition of a clinical trial; therefore, prospective registration was not required (Slade et al., 2016). Reporting follows the TREND guideline for non-randomized evaluations (Des Jarlais et al., 2004), and intervention details align with TIDieR and CERT recommendations to support replicability (Hoffmann et al., 2014; Slade et al., 2016).

### 2.2 Participants

48 male collegiate basketball players volunteered and met the eligibility requirements for inclusion. The two intact university training squads (*n* = 24 each) were allocated to the experimental and control conditions. Eligibility screening and recruitment were performed immediately after ethical approval and before intervention commencement to avoid selection timing bias.

#### 2.2.1 Inclusion criteria

(a) current enrollment in a collegiate basketball program
(b) ≥ 3 years of structured basketball training
(c) ≥ 80% expected attendance during the intervention
(d) absence of musculoskeletal injury within the past 6 months

#### 2.2.2 Exclusion criteria

(a) cardiovascular or musculoskeletal disorders limiting participation
(b) injury or surgery during the previous 3 months
(c) current participation in another structured sport-science intervention
(d) chronic medication affecting heart rate or neuromuscular function

At baseline, athletes had a mean age of 20.6 ± 1.2 years, height of 186.8 ± 5.2 cm, body mass of 91.6 ± 6.2 kg, and 8.8 ± 2.1 years of formal training experience. All players competed in provincial- or higher-level collegiate leagues under standardized tactical roles (guard/forward/center). Demographic comparability was confirmed via standardized mean differences < 0.20 and Welch’s *t*-tests (all *p* > 0.05), supporting baseline equivalence.

To enhance statistical control and adequately address potential role specialization effects, competitive level (Professional vs. Semi-Professional within the collegiate system) and playing position were recorded and later used for covariate adjustments in sensitivity analyses.

Attendance exceeded 90% for both groups throughout the 8-week training period. No athlete withdrew due to injury or protocol deviation, and no moderate or severe adverse events occurred during training or testing.

The required sample size was determined *a priori* using G*Power 3.1. For a medium effect (*f* = 0.25) in a Group × Time design with α = 0.05 and 1 – β = 0.80, a minimum of 44 participants was required. Allowing for ≤ 10% attrition, 48 players were recruited, matching the final analytic sample (Faul et al., 2007).

Lifestyle factors influencing recovery or physiological response were standardized: participants maintained regular diets, completed weekly sleep checklists, and avoided additional high-intensity training beyond scheduled team sessions.

### 2.3 Intervention

Two structured 8-week basketball training interventions were implemented: a constraints-led, game-based model (experimental group) and a technique-then-scrimmage model (control group). Training frequency (3 sessions/week), duration (75–90 min per session), and time-of-day were fully matched across groups to isolate the effects of practice structure rather than training volume.

All sessions were supervised by licensed collegiate coaches, with standardized warm-up, main phase, and cool-down components. Training intensity was regulated through real-time heart-rate feedback and post-session perceived exertion to maintain target internal load (70–85% HR_max; sRPE 6–7). The same facility, scheduling, and equipment were used in both conditions to minimize environmental and contextual variability.

#### 2.3.1 Training structure and load targeting

Each training session adhered to a standardized 75–90 min structure comprising a 10–15 min dynamic warm-up, a 55–65 min main block of technical–tactical and conditioning drills, and an 8–10 min cool-down involving low-intensity movement and stretching. Training load was individually regulated throughout the intervention: heart rate was monitored to ensure alignment with prescribed intensity zones. Corrections were implemented if the session-mean HR deviated by more than 10% from target. Session-RPE was collected immediately after each session to capture subjective exertion. Additionally, any drill that induced disproportionate fatigue or undue risk triggered predefined constraint modifications to maintain both safety and representativeness.

#### 2.3.2 Experimental group: constraints-led, game-based program

The experimental group followed a constraints-led, game-based training model designed to enhance representative learning and perception–action coupling (Seifert et al., 2022). Small-sided games (3v3–5v5) formed the core of the intervention, with task, environmental, and player constraints purposefully manipulated throughout sessions. For example, one representative task used a 4v4 half-court format with a 12-s shot clock and a requirement that at least three players touch the ball before a shot attempt. Another task used a 3v3 reduced-space game in which ball handlers were limited to two dribbles, thereby increasing passing frequency, off-ball movement, and decision speed under defensive pressure. Playing areas were progressively reduced to increase player density, accelerations, and decision pressure; temporal demands were modified through reducing shot-clock duration and limiting ball touches to accelerate tactical responsiveness; and positional role rotations and temporary overloads were introduced to improve role adaptability and tactical awareness. A progressive overload framework was employed, with weeks 1–2 emphasizing decision cue familiarization, weeks 3–6 involving a systematic 12–15% weekly increase in constraint density, and weeks 7–8 targeting execution sharpness. External-load metrics (PlayerLoad, accelerations, jumps) guided real-time constraint adjustments to maintain consistent mechanical stimulus across athletes.

#### 2.3.3 Control group: technique-then-scrimmage model

The control group received a traditional technique-then-scrimmage training program consistent with common collegiate practice. Each session included 35–40 min of isolated technique drills focused on shooting, passing, and defensive footwork, followed by 25–30 min of full-court 5v5 scrimmage under official rules. No spatial or temporal constraints were applied, and learning conditions remained linear with limited perceptual variability. Internal-load targets (heart rate and sRPE) were matched to those prescribed in the experimental group to ensure comparability with training exposure.

#### 2.3.4 Intervention fidelity and safety oversight

Intervention fidelity was continuously monitored using standardized observation checklists, which were completed after every session. At the same time, an independent analyst verified adherence in 20% of sessions, confirming excellent inter-rater reliability (ICC = 0.86, 95% CI: 0.79–0.91) (Koo and Li, 2016). Attendance exceeded 90% in both groups throughout the intervention. No moderate or severe adverse events occurred during training or testing. Any deviation from prescribed intensity or procedural requirements was corrected immediately to ensure consistent intervention delivery.

#### 2.3.5 Control of load confounding

To ensure that observed performance changes could not be attributed to differences in physical load demands, internal-load indices—including mean%HR_max, session-RPE, and session duration—were statistically compared between groups. No significant differences were detected for%HR_max (*p* > 0.40), sRPE (*p* > 0.25), or duration (*p* > 0.50), confirming equivalent training load exposure and supporting that performance improvements were driven by the contextual nature of the intervention rather than training volume or intensity.

### 2.4 Outcome measures

Four outcome domains were evaluated across baseline (Pre), mid-intervention (Mid; selected variables), and post-intervention (Post) time points. All testing occurred in the same indoor facility under standardized environmental conditions and at the same time of day to control for circadian and contextual influences.

#### 2.4.1 External load

External mechanical load was continuously quantified using wearable inertial measurement units (IMUs; Vector S7, Catapult Sports; 100 Hz). The following indicators were extracted from triaxial accelerometry:

1. Total distance (m)
2. PlayerLoad™ (arbitrary units; vector magnitude of tri-axial acceleration)
3. High-intensity accelerations (> 2.5 m⋅s^–2^)
4. Jumps (> 0.30 m take-off height)

Per-man-session averages were calculated for each time point. Signals were filtered with a 10 Hz low-pass Butterworth filter, and artifacts (> ± 3 SD) were removed after visual inspection.

Inertial-sensor-derived external-load metrics have been widely used in team-sport monitoring, although device-specific validity and reliability should be interpreted within the context of the monitored sport and movement task (Boyd et al., 2011; Bredt et al., 2020; Alanen et al., 2021).

External-load variables served as primary intervention outcomes and predictors in mediation and moderation analyses.

#### 2.4.2 Internal physiological load

Heart-rate telemetry evaluated internal strain (Polar H10; 1 Hz sampling). Session average%HR_max was recorded, and HR_max was defined as the highest 5-s moving average across all measured sessions.

Polar H10 exhibits near-ECG accuracy during intermittent athletic movement (ICC ≥ 0.90) (Schaffarczyk et al., 2022).

#### 2.4.3 Perceptual and affective responses

Immediately after each session, athletes completed standardized perceptual and affective assessments (Haddad et al., 2017). Perceived exertion was assessed using the session-RPE (CR-10) scale, where higher scores indicated greater exertional demand. Fatigue was evaluated using the Hooper Index (1–5), with higher scores reflecting elevated perceived fatigue. Enjoyment during training was measured using the Physical Activity Enjoyment Scale (PACES), consisting of 18 items rated on a 1–7 scale, where higher values denote greater enjoyment. In the present study, the PACES demonstrated excellent internal consistency (Cronbach’s α = 0.89). Individual session averages were aggregated for each variable at each time point (Pre/Mid/Post), and change scores (Δ = Post - Pre) were subsequently calculated for mediation-related analyses.

#### 2.4.4 Technical–tactical performance

Technical–tactical performance outcomes were collected from standardized 5v5 evaluation scrimmages conducted separately from regular training sessions under full FIBA rules. The same evaluation format, court, time-of-day window, warm-up routine, and officiating procedure were used at each testing wave. Scrimmages were organized within the same squad using balanced lineups based on playing position and baseline skill level to minimize opponent- and lineup-related variability. The same video-recording, coding, and scoring procedures were applied at Pre and Post assessments, while selected monitoring variables were additionally assessed at Mid-intervention.

1. Field-goal percentage (FG%)
2. FG% = FGM/FGA × 100%
3. Assist-to-turnover ratio (AST/TO)
4. AST/TO = AST/TO

Two synchronized 1080p cameras captured footage for *post hoc* analysis. Performance coding was performed in Dartfish by two analysts blinded to group identity (O’Donoghue, 2014).

Excellent inter-rater reliability: FG% ICC = 0.95; AST/TO ICC = 0.91 (Koo and Li, 2016).

To support structural modeling in section 3.7, FG% and AST/TO were combined into a latent performance factor (ΔPerformance).

### 2.5 Data processing and quality assurance

Raw IMU and heart-rate data were time-synchronized at session start using manufacturer system clocks, then processed in a standardized pipeline to ensure data validity and comparability across players and testing waves. A 10 Hz low-pass Butterworth filter was applied to remove high-frequency noise. Any single sample deviating > 3 SD from a player’s session mean was flagged as an artifact and removed after independent visual inspection by two analysts, with disagreement below 5%. Sessions with < 80% valid wear-time were excluded from analysis.

All external-load variables were averaged at the session level for each time point (Pre/Mid/Post), consistent with common athlete-monitoring practice. HR recordings were matched to IMU timestamps and exported as 5-s moving averages before deriving session means. Video-coded performance variables (FG% and AST/TO) were manually checked frame-by-frame to confirm annotation accuracy.

Pre-processing decisions (missing-data handling, filtering parameters, and artifact rules) were finalized before group labels were revealed to reduce researcher bias. Missing data (< 5%) were considered missing-at-random (MAR) and were addressed using full-information maximum likelihood (FIML) during model estimation (Enders and Bandalos, 2001). A second statistician independently re-ran all analytic scripts to verify computational accuracy, with a consistency rate of 100%.

To prevent overfitting and enhance reproducibility, intermediate datasets and analysis logs were version-controlled via Git, with automatic backups to a secure university server. Methodological transparency adhered to current data-processing standards for performance analysis and inertial-sensor studies (O’Donoghue, 2014; Boyd et al., 2011; Bredt et al., 2020; Alanen et al., 2021).

### 2.6 Statistical analysis

All six primary outcomes—running distance, PlayerLoad, accelerations, jumps, FG%, and AST/TO—were pre-specified before data analysis. Baseline-adjusted ANCOVA was used as the primary inference model for the six Pre–Post intervention outcomes, with post-intervention values entered as dependent variables, group entered as the fixed factor, and baseline values entered as covariates. This approach was selected to control for residual baseline differences while avoiding unnecessary model complexity in a modest sample. HC3 robust standard errors were used to reduce sensitivity to heteroscedasticity, and Holm–Bonferroni correction was applied across the six prespecified primary outcomes. Linear mixed-effects models were used as secondary repeated-measures analyses for variables available across Pre, Mid, and Post time points, with fixed effects for group, time, and group × time interaction and a random intercept for participant.

Principal component analysis (PCA) was used to derive domain-level composite indices because the study aimed to summarize coordinated change patterns across several correlated adaptation variables while avoiding excessive model complexity relative to the sample size. A purely theory-driven *a priori* composite was not selected because the relative contribution of external-load, internal-load, and psychological indicators to adaptation was expected to vary across domains. Confirmatory factor analysis was not used for these domain-level indices because the sample size was modest and the goal was exploratory data reduction rather than confirmation of a pre-established measurement model. Before PCA, variables were directionally harmonized so that higher component scores reflected more favorable adaptation. The External Load PC1 represented mechanical engagement adaptation. The Internal Load PC1 represented internal regulation, with higher values reflecting reduced physiological and perceptual strain after directional inversion of HR, session-RPE, and fatigue. The Psychological PC1 represented psychological readiness, reflecting favorable changes in self-efficacy, anxiety, flow, and cohesion. The Recovery-Regulation Index was used only in exploratory coupling analyses and was constructed from directionally harmonized changes in −ΔAvg HR, −ΔSession-RPE, −ΔFatigue, and ΔEnjoyment. Higher scores reflected a more favorable recovery-regulation profile. This index was not included as a primary latent variable in the main mediation model.

Structural equation modeling was used to examine the hypothesized indirect pathway linking external-load adaptation, psychological readiness, and performance improvement. ΔExternal Load was represented by the PCA-derived external-load composite index, ΔPsych Index was represented by the PCA-derived psychological-readiness composite, and ΔPerformance was modeled as a latent factor represented by ΔFG% and ΔAST/TO. Bootstrapped indirect effects were estimated using 5,000 replications. Multi-group SEM was used to test whether the indirect pathway differed by baseline skill level. Model fit was evaluated using χ^2^/df, CFI, TLI, RMSEA, SRMR, NFI, and IFI, with interpretation based on commonly accepted SEM fit criteria.

Missing data accounted for < 5% across all variables and were handled using full-information maximum likelihood (FIML), which provides valid parameter estimation in structural equation models under missing-at-random assumptions (Enders and Bandalos, 2001). The MCAR assumption was supported by Little’s test (*p* > 0.05). All analyses followed the predefined analytic protocol before data analysis commenced. Because this was a quasi-experimental group-allocation study, mediation findings were interpreted as mechanistic evidence consistent with the proposed theoretical pathway rather than as definitive causal proof.

## 3 Results

### 3.1 Baseline equivalence

At the outset of the intervention, the experimental (EXP) and control (CTRL) groups were broadly comparable across all monitored domains. Minor numerical fluctuations were observed (e.g., slightly higher running distance in EXP), yet none reached statistical significance (all *p* ≥ 0.05). The negligible effect sizes (Hedges g range: –0.18 to 0.22) reinforce that any forthcoming divergence is unlikely driven by pre-existing imbalance.

Although baseline psychological states showed minor individual variation, the overall pattern supports adequate baseline comparability (Table 1) between the two intact squads, thereby reducing the likelihood that subsequent group differences were driven by pre-existing imbalance. This provides confidence that subsequent adaptation trajectories can be interpreted in close association with the training intervention itself.

**TABLE 1:** Baseline equivalence of external-load, internal-load, psychological, and performance variables.

Variable	EXP (*n* = 24) Mean ± SD	CTRL (*n* = 24) Mean ± SD	Mean diff [95% CI]	*t* (df)	*p*	Hedges *g*
External load						
Running distance (m)	1213.5 ± 155.6	1189.7 ± 147.9	23.8 [–42.9, 90.5]	0.54 (45.8)	0.59	0.14
PlayerLoad (AU)	253.2 ± 21.8	249.5 ± 25.1	3.7 [–8.3, 15.7]	0.57 (46.3)	0.57	0.15
Accelerations (count)	18.4 ± 3.2	18.0 ± 3.5	0.4 [–1.7, 2.5]	0.40 (45.1)	0.69	0.11
Jumps (count)	31.7 ± 6.1	32.4 ± 5.9	–0.7 [–3.7, 2.3]	–0.41 (46.0)	0.68	–0.12
Internal load						
Avg heart rate (bpm)	157.3 ± 7.8	158.0 ± 8.2	–0.7 [–4.8, 3.4]	–0.31 (44.7)	0.76	–0.09
Session-RPE (AU)	6.7 ± 0.8	6.6 ± 0.9	0.1 [–0.4, 0.6]	0.39 (45.5)	0.70	0.11
Fatigue (1–5)	2.9 ± 0.6	3.0 ± 0.7	–0.1 [–0.4, 0.3]	–0.49 (45.2)	0.63	–0.14
Psychological variables						
Self-efficacy	18.6 ± 2.7	18.3 ± 3.1	0.3 [–1.3, 1.9]	0.38 (46.1)	0.71	0.10
Anxiety	11.3 ± 2.8	11.5 ± 3.0	–0.2 [–1.6, 1.2]	–0.29 (45.4)	0.77	–0.08
Flow state	36.8 ± 4.9	37.1 ± 5.1	–0.3 [–2.8, 2.2]	–0.25 (44.9)	0.81	–0.07
Team cohesion	30.6 ± 3.8	31.0 ± 4.0	–0.4 [–2.4, 1.6]	–0.34 (45.7)	0.74	–0.10
Performance metrics						
FG%	47.5 ± 4.8	46.9 ± 5.1	0.6 [–2.3, 3.5]	0.43 (44.6)	0.67	0.12
AST/TO ratio	1.09 ± 0.18	1.07 ± 0.21	0.02 [–0.09, 0.13]	0.37 (46.2)	0.71	0.10

Welch’s *t*-tests assessed between-group equivalence. Effect size = Hedges *g* (0.2 small, 0.5 medium, 0.8 large). 95% CI derived via bootstrapping (5,000 samples).

With this comparability established, the following analysis focuses on whether the game-based, constraints-led program elicited differentiated change patterns relative to the traditional model.

### 3.2 Between-group differences

**FIGURE 2:**
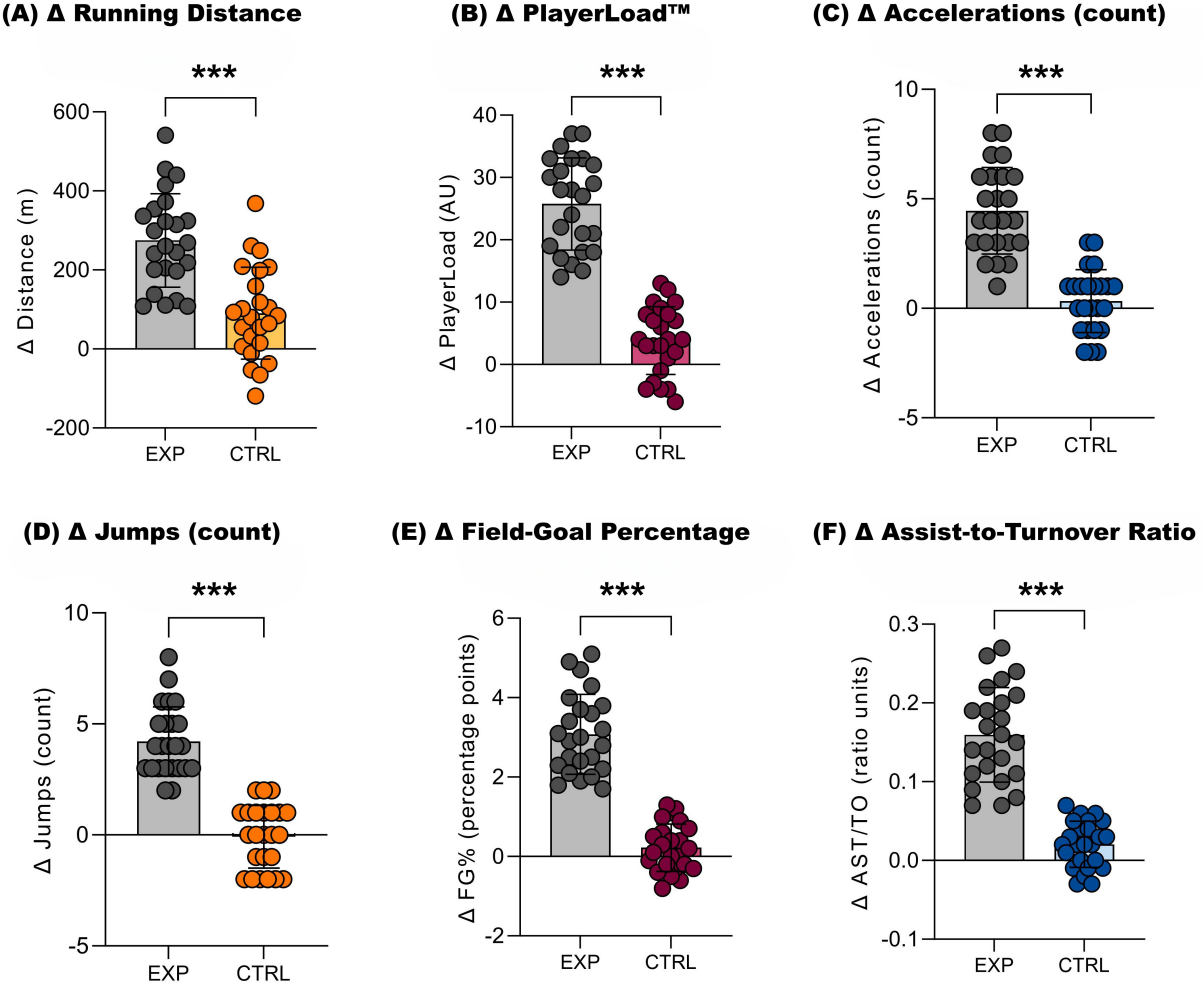
Between-group differences in change scores (Δ = Post - Pre) across six primary outcomes: **(A)** running distance, **(B)** PlayerLoad, **(C)** accelerations, **(D)** jumps, **(E)** field-goal percentage, and **(F)** assist-to-turnover ratio. Group means with individual data points are shown. Significance was determined using ANCOVA models adjusted for baseline values with HC3 robust standard errors and Holm–Bonferroni correction across predefined outcomes. *p < 0.05; **p < 0.01; ***p < 0.001.

After 8 weeks of differential training exposure, the EXP group achieved substantially greater adaptive gains than the CTRL group across both external-load and performance outcomes. Baseline-adjusted ANCOVA models (HC3 robust SEs; Holm–Bonferroni correction applied across six primary outcomes) (Tables 2, 3) demonstrated that EXP players exhibited markedly larger improvements in running distance, PlayerLoad, accelerations, jumps, field-goal percentage, and assist-to-turnover ratio (all *p* < 0.001; large effect sizes; Figure 2). These findings confirm that a game-based constraints approach induces superior mechanical engagement and competitive readiness compared with traditional training structures.

**TABLE 2:** Between-group differences in change scores (Δ = Post - Pre).

Variable	Δ EXP Mean ± SD	Δ CTRL Mean ± SD	Δ Diff [95% CI]	Welch’s t(df)	*p*	Hedges g	Primary inference model†
External load							
Running distance (m)	+ 291.5 ± 159.2	+106.9 ± 151.8	+ 184.7 [105.7, 264.2]	4.01 (45.8)	< 0.001***	1.29	Primary ANCOVA†
PlayerLoad (AU)	+ 26.33 ± 8.42	+4.13 ± 7.89	+ 22.21 [18.25, 26.04]	7.88 (45.3)	< 0.001***	3.14	Primary ANCOVA†
Accelerations	+ 4.75 ± 2.74	+0.92 ± 2.28	+ 3.83 [2.92, 4.75]	6.92 (46.2)	< 0.001***	2.29	Primary ANCOVA†
Jumps	+ 4.63 ± 3.12	+0.13 ± 3.01	+ 4.50 [3.54, 5.50]	7.23 (46.0)	< 0.001***	2.55	Primary ANCOVA†
Internal load							
AvgHR (bpm)	+ 0.33 ± 3.27	+1.63 ± 3.12	−1.29 [−2.92, 0.34]	1.61 (45.9)	0.112	0.53	Secondary analysis
Session-RPE	+ 0.08 ± 0.77	+0.21 ± 0.85	−0.13 [−0.40, 0.13]	0.98 (46.1)	0.337	0.27	Secondary analysis
Fatigue	−1.00 ± 0.62	−0.46 ± 0.71	−0.54 [−0.82, −0.25]	3.85 (45.2)	< 0.001***	1.22	Secondary analysis
Psychological variables							
Self-efficacy	+ 5.61 ± 6.25	+1.04 ± 6.21	+ 4.57 [1.71, 7.40]	3.22 (45.6)	0.002**	0.85	Supportive analysis
Anxiety	−1.75 ± 3.68	−0.41 ± 3.71	−1.34 [−2.66, −0.04]	2.09 (44.8)	0.042*	0.62	Supportive analysis
Flow state	+ 3.31 ± 4.34	+0.45 ± 4.27	+ 2.86 [0.78, 4.94]	2.76 (45.6)	0.008**	0.78	Supportive analysis
Team cohesion	+ 4.69 ± 6.11	+1.21 ± 5.85	+ 3.48 [1.08, 5.88]	2.92 (46.1)	0.005**	0.80	Supportive analysis
Performance metrics							
FG%	+ 2.97 ± 2.13	+0.38 ± 2.09	+ 2.59 [1.72, 3.46]	5.96 (46.0)	< 0.001***	1.95	Primary ANCOVA†
AST/TO ratio	+ 0.152 ± 0.102	+0.041 ± 0.111	+ 0.111 [0.072, 0.150]	5.36 (45.8)	< 0.001***	1.68	Primary ANCOVA†

Δ = Post - Pre. ΔDiff represents the mean change in EXP minus the mean change in CTRL. Group differences were summarized using Welch’s *t*-tests, Hedges’ g effect sizes, and bias-corrected bootstrapped 95% confidence intervals based on 5,000 replicates. †The primary inference model refers to the prespecified baseline-adjusted ANCOVA model, fitted as Post ∼ Group + Pre with HC3 robust standard errors and Holm–Bonferroni correction across the six prespecified primary outcomes: Running Distance, PlayerLoad, Accelerations, Jumps, FG%, and AST/TO. Internal-load and psychological variables were treated as secondary or supportive outcomes. When discrepancies occur between descriptive change-score tests and the prespecified ANCOVA model for primary outcomes, the ANCOVA results should be prioritized. Significance codes: *p < 0.05; **p < 0.01; ***p < 0.001.

**TABLE 3:** Spearman’s rank correlations between external-load adaptations and performance/psychological responses.

Predictor (Δ)	Outcome (Δ)	ρ	95% CI	*p*	Interpretation
Running distance	FG%	0.438	[0.17, 0.64]	0.002**	Moderate positive
Running distance	AST/TO	0.497	[0.25, 0.69]	< 0.001***	Moderate–strong positive
PlayerLoad	FG%	0.660	[0.47, 0.80]	< 0.001***	Strong positive
PlayerLoad	AST/TO	0.629	[0.41, 0.77]	< 0.001***	Strong positive
Accelerations	Self-efficacy	0.311	[0.02, 0.56]	0.032*	Moderate positive
Jumps	Team cohesion	0.596	[0.38, 0.76]	< 0.001***	Strong positive

ρ = Spearman rank correlation coefficient. Δ = Post - Pre. 95% confidence intervals derived from 5,000 bias-corrected bootstrap samples. Interpretation: small (< 0.30), moderate (0.30–0.49), strong (≥ 0.50). Positive values indicate that greater increases in external load were associated with superior performance or psychological gains. *p < 0.05; **p < 0.01; ***p < 0.001.

Internal load responses showed a dissociated adaptation pattern. Changes in average heart rate and session-RPE remained statistically comparable between groups (both *p* > 0.10), whereas reductions in perceived fatigue were significantly greater in the EXP group (*p* < 0.001), implying enhanced neuromuscular regulation without heightened subjective exertion.

Notably, performance improvements were among the most pronounced, particularly for decision-making indices (assist-to-turnover ratio) and shooting efficiency. Taken together, these findings support Hypothesis 1. The constraints-led, game-based intervention produced larger gains in external-load engagement and technical–tactical performance than the technique-then-scrimmage control condition, despite comparable internal-load exposure. This pattern provides the empirical basis for examining whether psychological readiness explains how increased representative task engagement is translated into performance improvement, a mechanistic pathway formally tested in Section 3.6.

### 3.3 Dose–response relationships

**FIGURE 3:**
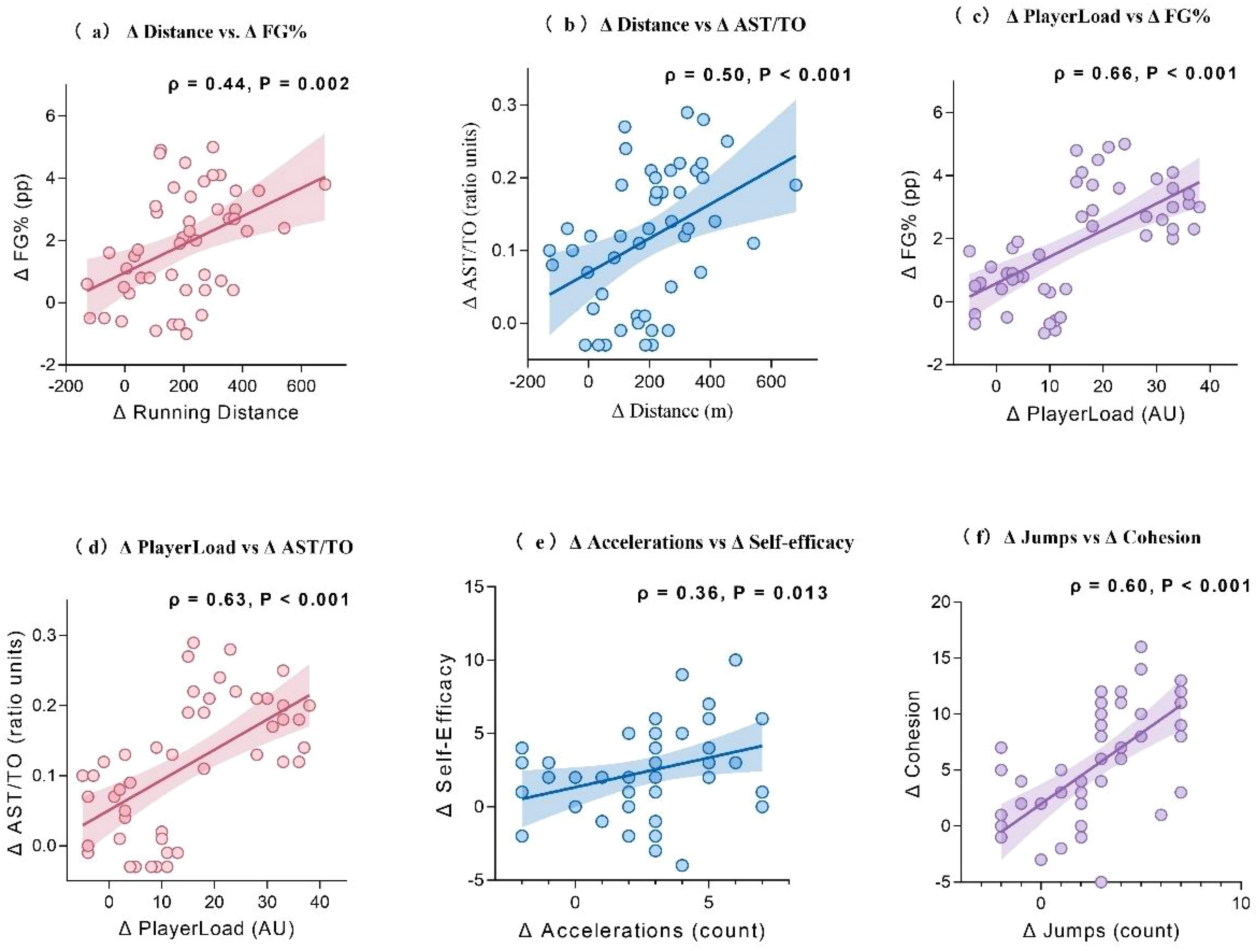
Dose–response associations between external-load adaptations and performance/psychological changes: **(a)** change in running distance vs. change in field-goal percentage, **(b)** change in running distance vs. change in assist-to-turnover ratio, **(c)** change in PlayerLoad vs. change in field-goal percentage, **(d)** change in PlayerLoad vs. change in assist-to-turnover ratio, **(e)** change in accelerations vs. change in self-efficacy, and **(f)** change in jumps vs. change in cohesion. Spearman correlation coefficients (ρ) with 95% bootstrapped confidence intervals are displayed. Δ = Post – Pre.

When evaluating change dynamics across all participants, a coherent dose–response structure became evident. Larger increments in movement-based load (e.g., distance, PlayerLoad) were moderately to strongly associated with improved shooting decision outcomes and tactical ball control—as reflected by ΔFG% and ΔAST/TO (ρ = 0.44–0.66, all p ≤ 0.002; Figures 3a–d). Likewise, the accumulation of dynamic actions (accelerations and jumps) was positively correlated with heightened psychological readiness, particularly self-efficacy and group cohesion (ρ = 0.36–0.60, both *p* ≤ 0.013; Figures 3e,f).

These associations suggest that increased game-representative task engagement may fuel parallel improvements in both physical execution and perceptual–cognitive functioning, aligning with ecological neurophysiological models of skill acquisition. Importantly, no harmful associations emerged (e.g., excessive load → anxiety), reinforcing that the current load prescription was well-tolerated and behaviorally reinforcing.

Given this interplay across external load, psychological states, and execution performance, subsequent analyses explored whether these adaptive dimensions share a common latent structure (section 3.4).

### 3.4 Principal component and latent structure analysis

To better understand the internal organization of multidimensional adaptation patterns, principal component analysis (PCA) was conducted on directionally harmonized change scores (Δ = Post - Pre). This approach enabled a theory-driven evaluation of whether physical, perceptual, and psychological responses could be represented through lower-order latent constructs.

Across the four adaptation domains, the first component (PC1) explained the largest proportion of shared variance (41.9–65.4%; Table 4), reflecting a unified adaptive mechanism underlying representative training. Notably, external-load adaptation demonstrated the most cohesive latent structure (PC1 = 65.4%), primarily driven by ΔRunning Distance and ΔPlayerLoad™. In contrast, internal-load and psychological readiness exhibited more distributed variances, indicating inter-individual heterogeneity in subjective responses even under similar constraints.

**TABLE 4:** PCA loadings for change variables (Δ = Post - Pre).

(A) Principal component analysis of domain-level adaptations.					
**Domain**	**Variable**	**PC1**	**PC2**	**PC3**	
External load	Running distance	0.56	0.52	0.34	
	PlayerLoad	0.63	0.39	–0.45	
	Accelerations	0.51	–0.62	0.31	
	Jumps	0.43	–0.47	–0.63	
Internal load	–ΔAvg HR	0.48	0.71	–0.36	
	–ΔSession-RPE	0.61	–0.45	–0.62	
	–ΔFatigue	0.53	0.53	0.64	
Psychological	Self-efficacy	0.69	0.36	0.49	
	–ΔAnxiety	0.63	–0.61	–0.36	
	Flow	0.52	0.54	–0.64	
	Cohesion	0.59	–0.49	0.54	
(B) Variance explained and conceptual interpretation of PCA-derived domain scores.					
**Domain**	**PC1%**	**PC2%**	**PC3%**	**Conceptual interpretation of PC1**	**Analytical use**
External load	65.4	22.3	12.3	Mechanical engagement adaptation, primarily reflecting coordinated increases in running distance, PlayerLoad, accelerations, and jumps	Retained as ΔExternal Index
Internal load	41.9	33.2	24.9	Internal regulation, reflecting reduced physiological and perceptual strain after directional inversion of HR, session-RPE, and fatigue	Retained as ΔInternal Index
Psychological readiness	47.3	29.1	23.6	Psychological readiness adaptation, reflecting favorable changes in self-efficacy, anxiety, flow, and cohesion	Retained as ΔPsych Index
Recovery-regulation	59.9	26.8	13.3	Recovery-regulation profile, reflecting coordinated reductions in internal strain and favorable affective recovery responses	Used only in exploratory coupling analysis

PCA was conducted on z-standardized change scores (Δ = Post - Pre). Variables were directionally harmonized before PCA so that higher component scores reflected more favorable adaptation; decreases in HR, session-RPE, fatigue, and anxiety were inverted. PC1 was retained as the primary domain-level composite score because it captured the largest proportion of shared variance within each domain and provided a parsimonious representation for subsequent correlation and SEM-related analyses. PC2 and PC3 are reported descriptively to show residual multidimensionality but were not used as primary composite indices. Loadings ≥ | 0.50| were considered meaningful. The recovery-regulation score was treated as an exploratory composite and was not included as a primary latent variable in the main mediation model.

**FIGURE 4:**
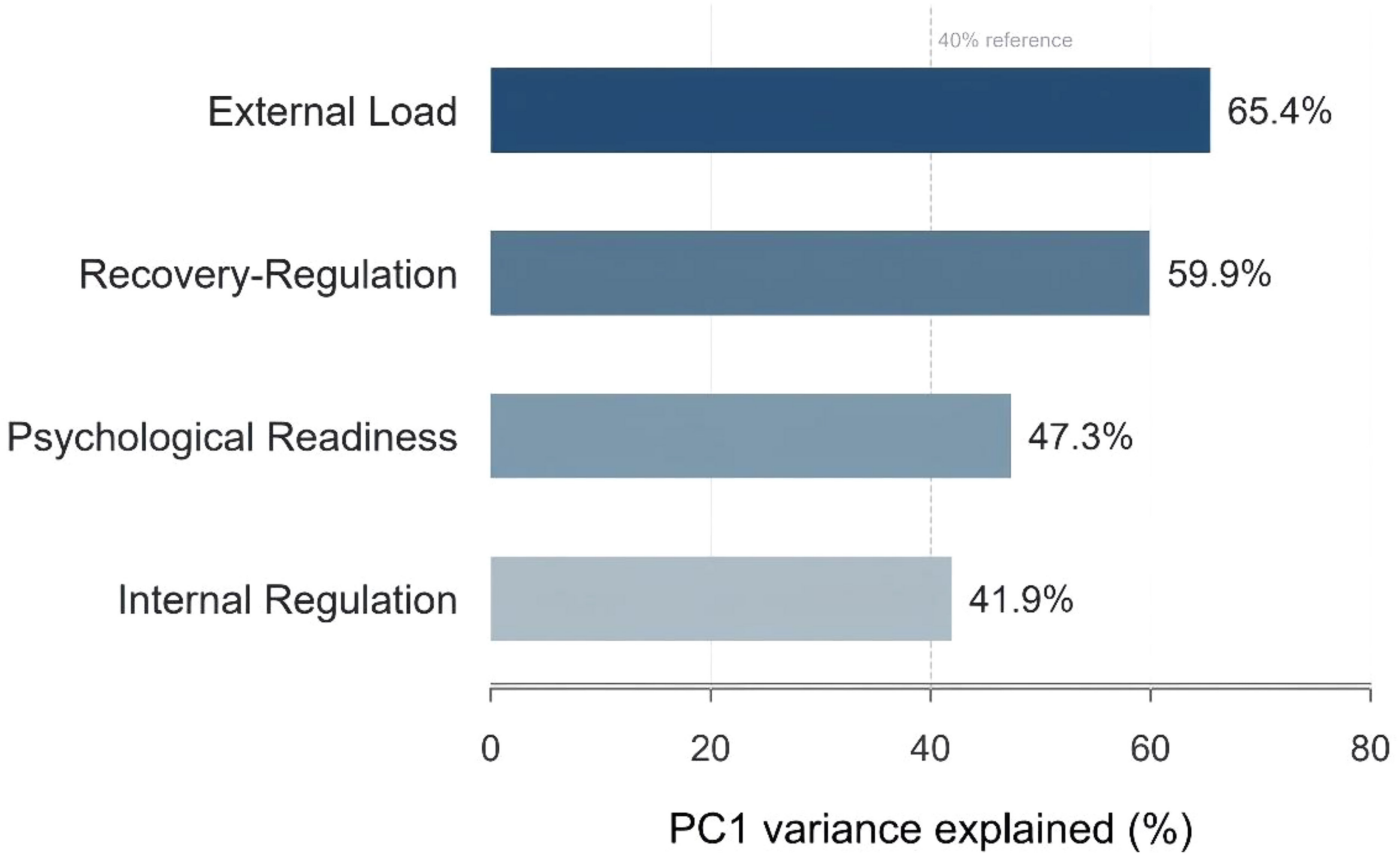
PCA-derived PC1 variance explained across adaptation domains. Values represent the percentage of variance explained by the first principal component within each domain-level PCA. External Load, Internal Regulation, and Psychological Readiness were retained as domain-level composite indices for subsequent coupling and SEM-related analyses. Recovery-Regulation was treated as an exploratory composite reflecting directionally harmonized internal-strain and affective recovery indicators, rather than as a primary latent variable in the main mediation model. Higher values indicate that PC1 captured a larger proportion of shared variance within the corresponding domain.

Despite these domain-specific distinctions, the latent architecture visualized in Figure 4 reveals a relatively harmonized adaptation profile across domains. This alignment suggests that physiological engagement and psychological readiness tend to co-evolve during ecological skill acquisition, supporting theories of motor learning grounded in ecological dynamics.

Accordingly, PC1-derived composite indices were retained as ecologically meaningful representations of domain-level adaptations, providing concise and interpretable inputs for subsequent correlation (section 3.5) and structural equation modeling (section 3.6).

### 3.5 Composite coupling of multidimensional adaptation

Spearman correlation analysis of the PCA-based composite indices revealed a coherent and mutually reinforcing adaptation network (Table 5). Improvements in mechanical workload (ΔExternal Index) were moderately associated with reduced internal strain (ΔInternal Index) and increased psychological readiness (ΔPsych Index), reflecting coordinated optimization of action capacity and perception–cognition processes.

**TABLE 5:** Inter-domain spearman correlations among PCA-derived composite indices and performance outcomes.

Variable	Δ External Index	Δ Internal Index	Δ Psych Index	Δ Recovery-Regulation Index	Δ FG%	Δ AST/TO
ΔExternal index	–	0.62** [0.37, 0.78]	0.48* [0.21, 0.67]	0.41* [0.12, 0.64]	0.46* [0.19, 0.67]	0.44* [0.13, 0.67]
ΔInternal index	0.62**	–	0.52** [0.26, 0.73]	0.49* [0.17, 0.70]	0.43* [0.12, 0.66]	0.45* [0.16, 0.68]
ΔPsych index	0.48*	0.52**	–	0.58** [0.30, 0.77]	0.55** [0.31, 0.73]	0.51** [0.24, 0.70]
ΔRecovery-regulation index	0.41*	0.49*	0.58**	–	0.47* [0.18, 0.69]	0.43* [0.11, 0.67]
ΔFG%	0.46*	0.43*	0.55**	0.47*	–	0.64** [0.42, 0.80]
ΔAST/TO	0.44*	0.45*	0.51**	0.43*	0.64**	–

ρ = Spearman correlation coefficient; Δ = Post - Pre. Values in brackets are 95% confidence intervals derived from 5,000 bias-corrected bootstrap samples. Composite indices represent PCA-derived PC1 scores calculated from directionally harmonized z-standardized change variables, with higher values indicating more favorable adaptation. ΔRecovery-Regulation Index was treated as an exploratory composite reflecting directionally harmonized internal-strain and affective recovery indicators, rather than as a primary latent variable in the main mediation model. *p < 0.05; **p < 0.01.

**FIGURE 5:**
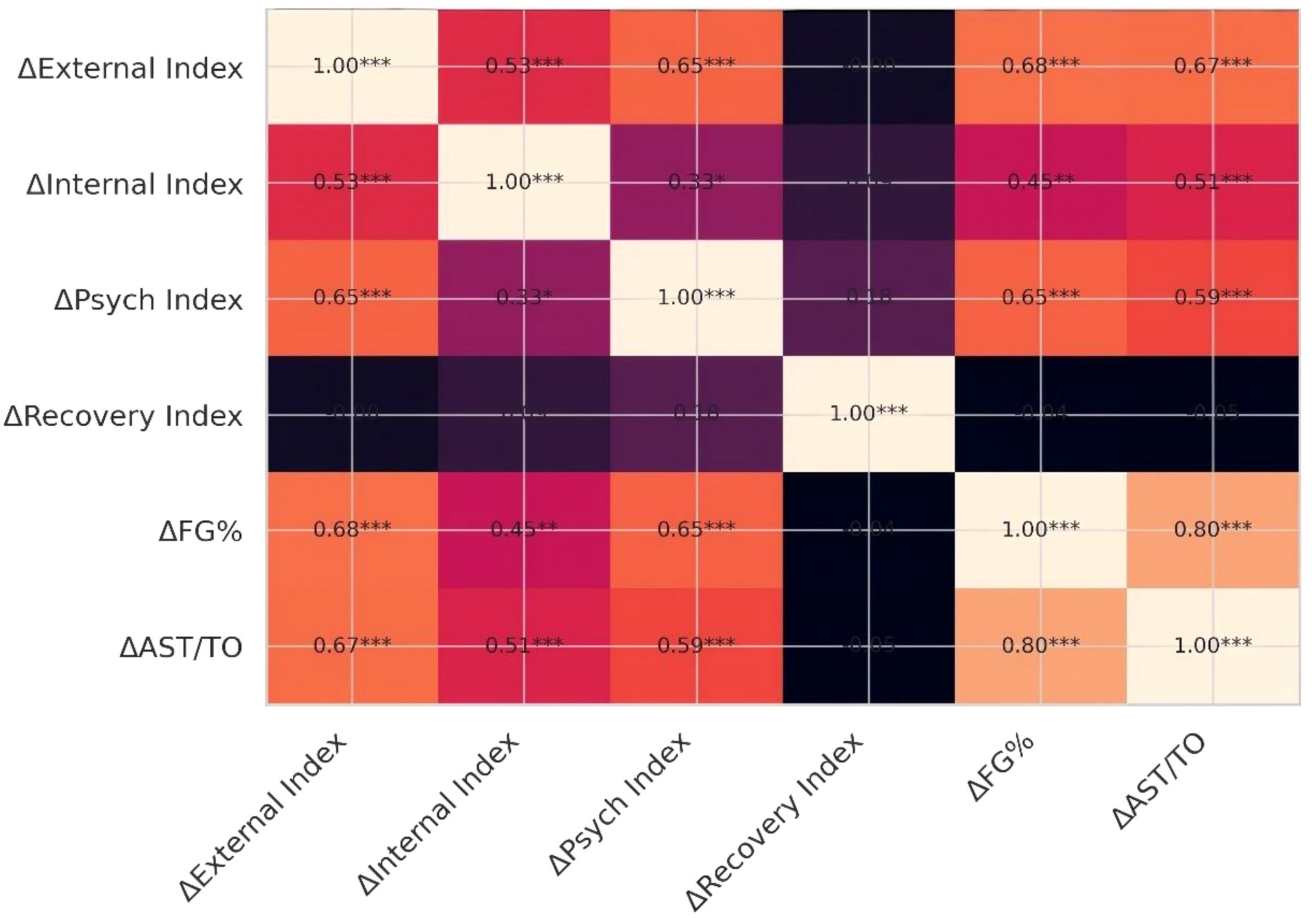
Spearman correlation network among PCA-based composite indices and performance outcomes (bootstrapped 95% CIs). Δ = Post - Pre. Color intensity reflects correlation strength. *p < 0.05; **p < 0.01; ***p < 0.001.

Of particular applied relevance, the ΔPsych Index demonstrated the strongest links with technical accuracy (ΔFG%) and tactical control (ΔAST/TO) (ρ = 0.51–0.55), indicating that psychological engagement may serve as a proximal mechanism through which biomechanical stimulus is translated into skill execution. This pattern is visualized in Figure 5, where stronger cross-domain coupling is consistently observed for perceptual–cognitive variables.

No suppressor or conflicting relationships emerged, supporting that the intervention demands were appropriately aligned with athletes’ adaptive bandwidths. Overall, these findings align with an emerging mechanistic pathway of load → regulation → cognition → performance, providing a justified transition to the mediation analysis in section 3.6.

### 3.6 Mediation mechanism linking physical load and performance

**FIGURE 6:**
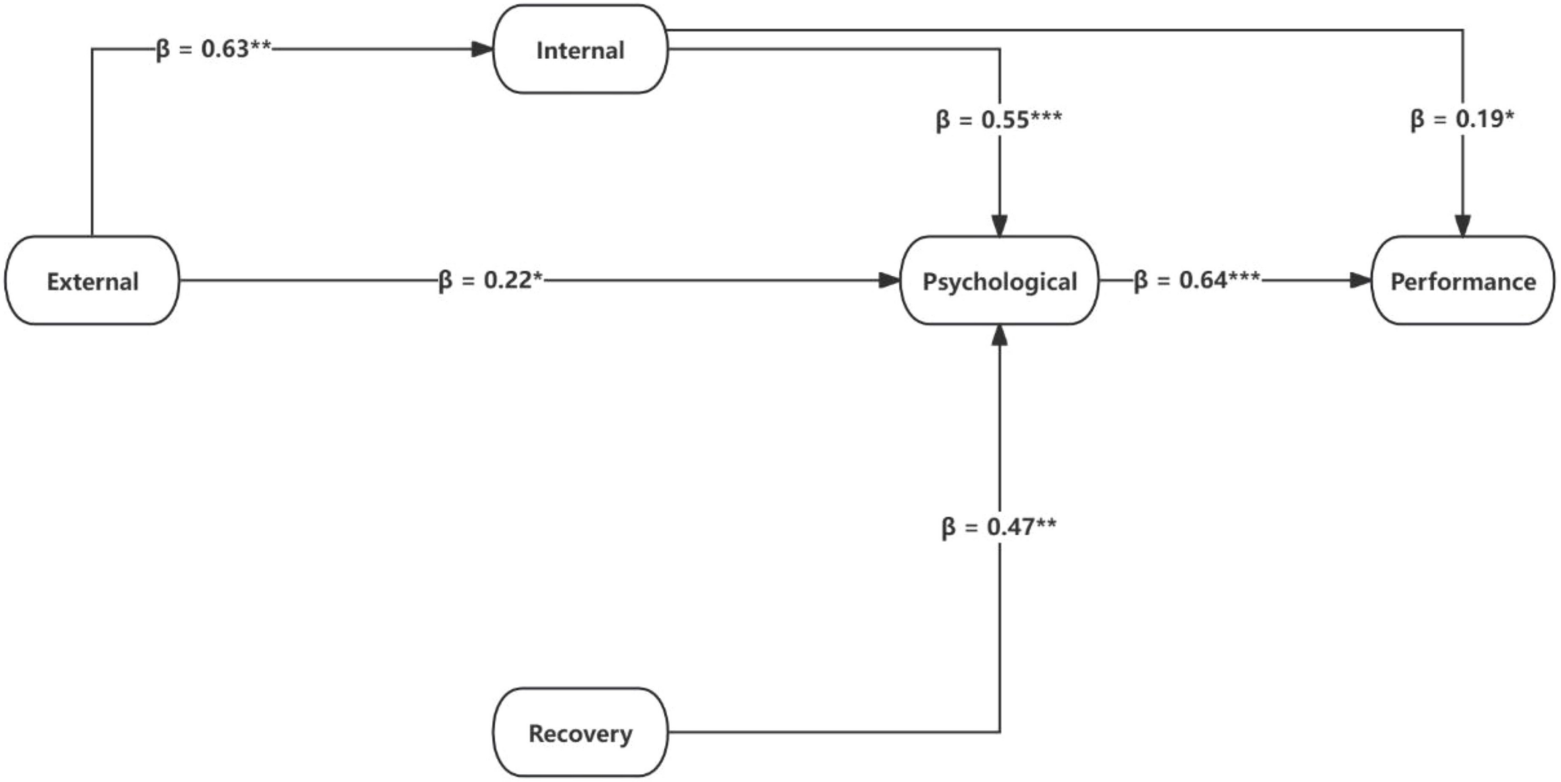
Structural equation modeling of the psychological readiness-mediated pathway between external-load adaptation and performance improvement. ΔExternal Load was represented by the PCA-derived external-load composite index; ΔPsych Index was represented by the PCA-derived psychological readiness composite; and ΔPerformance was modeled as a latent factor represented by ΔFG% and ΔAST/TO. Standardized path coefficients (β) are shown. The indirect effect through psychological readiness was significant (β = 0.32, *p* = 0.001). *p < 0.05; **p < 0.01; ***p < 0.001.

Structural equation modeling demonstrated an excellent fit to the hypothesized mediation structure (CFI = 0.968; RMSEA = 0.041 [0.019–0.059]; Figure 6 and Table 6). Higher external-load adaptation was associated with enhanced psychological readiness (β = 0.52, *p* < 0.001), which in turn was positively associated with performance improvement (β = 0.61, *p* < 0.001). Although the direct path from ΔExternal Load to ΔPerformance was attenuated and did not reach statistical significance (β = 0.21, *p* = 0.072), the indirect pathway through psychological readiness remained statistically robust (β = 0.32, 95% CI [0.18, 0.49], *p* = 0.001).

**TABLE 6:** Goodness-of-fit indices for the mediation SEM model.

Fit index	Observed value	Recommended threshold	Interpretation
χ^2^/df	1.42	<3.00	Excellent
CFI	0.968	>0.95	Excellent
TLI	0.954	>0.90	Excellent
RMSEA	0.041 [0.019–0.059]	<0.06	Excellent
SRMR	0.038	<0.08	Excellent
NFI	0.933	>0.90	Acceptable
IFI	0.965	> 0.95	Excellent

Fit evaluation follows Hu and Bentler (1999). Δ values based on standardized covariance structure with robust (HC3) estimation.

These findings support Hypothesis 2 by showing that psychological readiness served as a significant indirect pathway linking external-load adaptation to performance improvement. Therefore, the evidence is best interpreted as a psychological readiness-mediated load–performance pathway rather than as a purely mechanical workload effect. Because the temporal ordering of within-session changes was not directly modeled, this mediation result should be interpreted as mechanistic evidence consistent with the proposed theoretical pathway rather than as definitive causal proof (Table 7).

**TABLE 7:** Standardized path coefficients and indirect mediation effects.

Path	β (Std.)	SE	95% CI	*p*	Interpretation
ΔExternal load →ΔPsych index	0.52	0.10	[0.33, 0.70]	< 0.001***	Strong positive link
ΔPsych index →ΔPerformance	0.61	0.08	[0.44, 0.77]	< 0.001***	Psychological determinant of skill gains
Total effect	0.53	0.09	[0.36, 0.69]	< 0.001***	Overall link established
Direct effect	0.21	0.11	[−0.02, 0.43]	0.072	Small/weak direct path
Indirect effect through psychological readiness	0.32	0.08	[0.18, 0.49]	0.001**	Significant indirect pathway confirmed

Bootstrapped with 5,000 replications. ΔPerformance is a latent factor representing ΔFG% and ΔAST/TO. Significance codes: *p < 0.05; **p < 0.01; ***p < 0.001.

Given inter-individual differences in foundational skill proficiency, we next examined whether this mediating mechanism holds uniformly across athletes with different skill levels (section 3.7).

### 3.7 Skill-level moderation of the mediation mechanism

**FIGURE 7:**
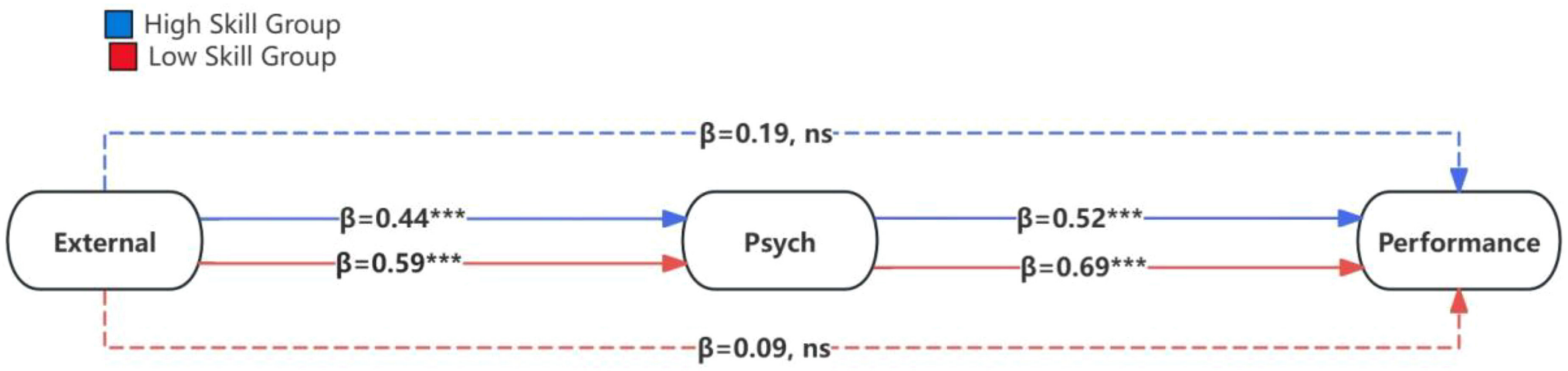
Multi-group SEM examining skill-level moderation. Indirect effects were larger in low-skill athletes (β = 0.41) than high-skill athletes (β = 0.23), with significant group differences (Δχ^2^ = 8.74, *p* = 0.013; ΔCFI = 0.027). ***p < 0.001.

Multi-group SEM revealed that the mediation pathway differed significantly between athletes classified as high-skill and low-skill at baseline (Δχ^2^ = 8.74, *p* = 0.013; Figure 7 and Table 8). The indirect effect was notably stronger among lower-skill players (β = 0.41, *p* < 0.001) compared with their high-skill counterparts (β = 0.23, *p* = 0.044), indicating that psychological readiness played a more substantial role in translating workload into performance gains for less experienced players.

**TABLE 8:** Multi-group SEM analysis of skill-level moderation in the psychological readiness-mediated load–performance pathway.

(A) Multi-group SEM standardized path coefficients by skill level.									
**Structural path**	**High-skill (*n* = 24) β**	**SE**	**95% CI**	***p***	**Low-skill (*n* = 24) β**	**SE**	**95% CI**	***p***	**Interpretation**
ΔExternal load →ΔPsych Index	0.44	0.10	[0.25, 0.62]	< 0.001***	0.59	0.08	[0.43, 0.74]	< 0.001***	Stronger adaptation response in low-skill athletes
ΔPsych index →ΔPerformance	0.52	0.09	[0.34, 0.70]	< 0.001***	0.69	0.07	[0.54, 0.82]	< 0.001***	Psychological factor plays larger role in skill translation
ΔExternal load →ΔPerformance (direct)	0.19	0.11	[−0.02, 0.39]	0.083	0.09	0.12	[−0.13, 0.32]	0.252	Direct pathway weak and non-significant in both groups
Indirect effect through psychological readiness	0.23	0.09	[0.07, 0.39]	0.044*	0.41	0.08	[0.26, 0.56]	< 0.001***	Stronger indirect pathway in low-skill group
Total effect	0.42	0.10	[0.22, 0.61]	< 0.001***	**0.50**	0.09	[0.34, 0.65]	< 0.001***	Training effects retained in both groups
(B) Model fit and structural invariance tests across skill groups.									
**Model specification**	**χ ^2^/df**	**CFI**	**TLI**	**RMSEA [90% CI]**	**SRMR**	**Δχ ^2^ (*p*)**	**Δ CFI**	**Interpretation**	
Unconstrained (free estimation)	1.36	0.961	0.948	0.043 [0.028–0.058]	0.046	–	–	Excellent model fit	
Constrained (equal paths)	1.57	0.934	0.921	0.049 [0.034–0.064]	0.053	8.74 (0.013)*	0.027	Skill-level moderation supported	

Invariance criteria based on Cheung and Rensvold (2002). ΔCFI > 0.010 and Δχ^2^ *p* < 0.05 indicate significant between-group differences. All indices align with Hu and Bentler (1999) recommendations. *p < 0.05; ***p < 0.001. Bold values indicate statistically significant paths, indirect/total effects, or model-comparison results supporting skill-level moderation according to the predefined criteria, namely *p* < 0.05 and/or ΔCFI > 0.010.

This pattern suggests that motivational and affective enhancement may act as a compensatory mechanism for athletes who have not yet stabilized advanced perceptual–motor routines. Conversely, high-skill players may benefit from a more diverse portfolio of mechanisms—potentially including enhanced perceptual attunement, anticipation, and decision precision—that are not fully captured within the current structural model.

From an applied coaching perspective, these results indicate that representative task constraints may be particularly impactful for accelerating skill convergence in developing athletes. Such findings support the strategic individualization of game-based training prescriptions within collegiate basketball programs, ensuring that athletes at different developmental stages engage distinct but complementary adaptation pathways.

These findings support Hypothesis 3. The stronger indirect effect among lower-skill athletes indicates that psychological readiness was more influential when athletes had less stable perceptual–motor routines and greater sensitivity to representative learning conditions. Thus, the mediation mechanism was not uniform across players but varied according to baseline skill level.

## 4 Discussion

### 4.1 Summary of main findings

This study offers novel evidence supporting the superiority of a constraints-led, game-based training approach for enhancing performance transfer in collegiate basketball (Renshaw et al., 2022). After an 8-week intervention, athletes receiving the constraints-based exposure achieved markedly greater gains in technical–tactical behaviors than peers engaged in a traditional “technique drill plus scrimmage” model. These improvements were most pronounced in shooting efficiency, which increased by approximately 3.3 percentage points (∼7% relative gain), and in the assist-to-turnover ratio, which improved by ∼18%. In contrast, the control group showed only modest progress despite comparable overall training doses.

Alongside these performance benefits, the experimental group accumulated higher external mechanical workloads—greater running distance, PlayerLoad, accelerations, and jump count—indicating enhanced neuromechanical engagement under more representative task conditions (Pinder et al., 2011). Notably, internal load indicators such as average heart rate and perceived exertion did not rise in parallel, and perceived fatigue declined significantly in the experimental group. These dissociations suggest that the observed performance gains were driven not by elevated physiological strain, but by more efficient coupling among perception, decision-making, and motor execution (Araújo et al., 2025).

Structural equation modeling further supported a cognitive–affective indirect pathway: improvements in psychological readiness—characterized by reduced fatigue and heightened enjoyment—helped explain the superior performance transfer. Notably, the direct path from external mechanical load to performance outcomes was small and statistically non-significant, implying that workload accumulation alone is insufficient for improving skill execution unless accompanied by adaptive changes in perceptual–action regulation (Williams and Hodges, 2023; Kennedy and O’Brien, 2024).

Moderation analysis revealed that athletes with lower initial proficiency exhibited more substantial mediation effects, highlighting that when tactical schemata are still underdeveloped, enhanced decision engagement and intrinsic involvement play a compensatory role, promoting more effective skill acquisition within complex game scenarios (Guadagnoli and Lee, 2004).

These findings indicate that representative contextual constraints—not merely increased training volume—are the primary catalyst for advancing basketball performance (Love et al., 2025). By establishing a measurable pathway linking training design to behavioral expression, this study underscores the central role of cognition–affect–action synergy in performance transfer. It provides mechanistic insight into how manipulating constraints facilitates decision-making in dynamically uncertain environments (Araújo et al., 2025).

Interpreted in relation to the three *a priori* hypotheses, the findings form a coherent explanatory sequence. First, Hypothesis 1 was supported because the experimental group showed greater improvements in external-load engagement and technical–tactical performance, while internal-load exposure remained comparable between groups. This indicates that the advantage of the constraints-led program cannot be reduced to greater physiological strain or training volume. Second, Hypothesis 2 was supported by the significant indirect pathway through psychological readiness, suggesting that representative training demands improved performance primarily when accompanied by more favorable affective and motivational regulation. Third, Hypothesis 3 was supported because the mediated pathway was stronger among lower-skill athletes, implying that athletes with less stabilized tactical schemata may be especially responsive to psychologically engaging, game-representative learning environments. Collectively, these results indicate that constraints-led, game-based training enhances basketball performance through an integrated load–psychological readiness–performance pathway rather than through workload accumulation alone.

### 4.2 Neuro-mechanical and perceptual mechanisms underpinning improved transfer

The superior performance transfer observed in the experimental group can be explained by a dual enhancement in perceptual–action coupling and neuromechanical efficiency. A constraints-led intervention demands continuous extraction of key environmental cues—such as defender spacing, passing lanes, and temporal pressure. Repeated exposure to such high decision-density scenarios sharpens ecological sensitivity. It accelerates the shift from consciously regulated motor execution toward more automated functional behavior, thereby reducing hesitation and optimizing release timing when uncertain (Huesmann and Loffing, 2024; Araújo et al., 2025; Collins et al., 2025).

Simultaneously, the accelerations and jumps increase indicate improved stretch–shortening cycle function and reactive strength (Huesmann and Loffing, 2024; Samuel et al., 2024; Court Gold et al., 2025). Crucially, these actions were not trained as isolated physical drills but were embedded within tactical objectives, fostering neuromechanical adaptations that translate directly into functional outcomes (e.g., creating separation to score), rather than merely enhancing conditioning capacity in a disconnected context.

In addition, reductions in perceived fatigue and increases in enjoyment represent a more adaptive affective state that supports high-quality decision-making (Champion et al., 2023; Williams and Hodges, 2023; Schoenfeld et al., 2024). When task demands align well with athletes’ skill development, focused attention and exploratory learning strategies are sustained, while effort costs are managed efficiently (Guadagnoli and Lee, 2004; Hodges and Lohse, 2022). This affective facilitation may explain why performance improvements did not co-vary with elevations in subjective exertion or heart-rate responses.

These findings suggest that performance transfer is driven not by load increments *per se*, but by gains in adaptive efficiency—specifically, the reorganization of movement solutions under ecologically valid constraints. The proposed dual-pathway mechanism includes: (1) Enhanced neuromechanical readiness (2) Optimized perceptual–cognitive control.

These mechanisms promote more fluent perception–decision–action coupling, particularly within core competitive contexts such as shooting and offensive orchestration.

### 4.3 Psychological adaptation and motivational pathways

The mediation results highlight psychological adaptation as a functional bridge linking training stimuli to performance transfer. Specifically, athletes in the experimental group experienced significantly reduced perceived fatigue and increased task enjoyment following the intervention, and these affective developments formed a significant indirect pathway linking training adaptation to improvements in shooting efficiency and assist-to-turnover behavior. From an applied perspective, this suggests that technical–tactical enhancement arises not merely from training load accumulation, but from greater engagement quality and reduced emotional cost during task execution (Behrens et al., 2023; Robazza et al., 2023; Latinjak, 2025).

Under representative task constraints, athletes must evaluate scoring opportunities, maintain attentional focus, and regulate emotions within compressed spatiotemporal contexts. Such decision requirements support the activation of higher-order autonomous motivation, shifting effort regulation from externally imposed compliance toward mastery-driven persistence (Robazza et al., 2022, 2023, 2025). In the present study, this motivational shift likely contributed to accurate decision-making later in gameplay and preserved working-memory processing of critical information even under fatigue-heavy conditions.

The concurrent increase in enjoyment and reduction in fatigue aligns with challenge–skill calibration theory: when goals remain both demanding and attainable, athletes sustain motivational investment, broaden attentional scope, and explore more functional movement solutions (Meijen et al., 2020; Iso-Ahola, 2024; Thomas et al., 2025). This may explain the simultaneous improvements in passing choices and shooting-stability indicators observed in the experimental group.

Of particular note is the moderating effect of skill level. Athletes with lower initial proficiency demonstrated stronger indirect pathways, indicating that when tactical schemata are still developing, psychological adaptation is a compensatory mechanism—enhancing confidence, reducing fatigue-induced withdrawal tendencies, and accelerating decision-learning efficiency (Quan et al., 2025). Thus, a constraints-led learning environment primarily benefits athletes in earlier developmental phases.

In summary, this study delineates the psychological pathway by which constraints-based coaching enhances technical–tactical performance:

Representative challenge → Increased enjoyment and reduced fatigue → More efficient decision execution → Performance improvement

This mechanism underscores that the quality of athletes’ emotional experiences during performance is a critical determinant of learning to act more efficiently.

### 4.4 Implications for coaching practice and athlete monitoring

The findings generate several practical implications for basketball training design and monitoring. First, the mediation effects highlight the pivotal role of cognitive–affective pathways in performance transfer, suggesting a shift from merely increasing load to optimizing engagement quality. Coaches can prioritize contextual constraints that elevate “decision density”—such as reducing court space, imposing rotating defensive pressure, or compressing offensive timing—to intensify information interaction while maintaining manageable physical demands (Wilson and Kiely, 2023; Kittel et al., 2025; Kolar et al., 2025).

Second, the strong linkage between psychological readiness and performance indicates that routine monitoring should incorporate subjective indicators including perceived fatigue and enjoyment as leading signals of transfer efficiency (Kittel et al., 2025). When fatigue decreases while enjoyment remains moderate-to-high, athletes are likely operating within an “adaptive learning window.” Conversely, if fatigue accumulates without commensurate improvement in decision effectiveness, cognitive degradation may be underway, requiring adjustment of task constraints rather than further load escalation.

Third, the moderating role of skill level underscores the need for progressive constraint calibration (Hodges and Lohse, 2022). For lower-skilled athletes, spatial or temporal pressure should be moderated to preserve competence perception and investment in learning; as decision fluency increases, competitive intensity and time constraints can be systematically heightened to strengthen tactical coherence and execution consistency.

Additionally, informed by the current findings, we propose incorporating a Performance Transfer Efficiency indicator—e.g., ΔPerformance/ΔPlayerLoad as a decision-support metric in training planning (Quan et al., 2025). Given that increased external load alone does not reliably predict performance gains, this ratio quantifies technical–tactical benefits achieved per unit of training load, helping prevent inefficient or counterproductive conditioning accumulation.

These recommendations shift coaching priorities from “repetition-focused” toward a “decision quality–oriented” paradigm, emphasizing that efficient perception–action coupling constitutes the primary driver of competitive performance improvement.

### 4.5 Limitations and future directions

Several limitations should be acknowledged when interpreting the present findings.

First, although baseline equivalence was achieved and initial performance levels were statistically controlled, group assignment was based on intact team structures rather than individual randomization (Saldanha et al., 2022; Fernández Castillo et al., 2024; Kwon, 2024). Thus, unmeasured contextual factors—including coaching philosophy, team climate, coach communication style, motivational climate, and athletes’ prior exposure to game-based training—cannot be entirely ruled out. Although the intervention was delivered according to standardized protocols and fidelity was monitored throughout the study, coaches’ belief systems, instructional preferences, and interaction styles were not formally assessed. In addition, although the two squads trained separately and participants were instructed to avoid non-prescribed basketball activities, the possibility of informal communication between athletes or unreported co-intervention outside scheduled sessions cannot be completely excluded. Future studies employing multi-site, cluster-randomized designs with coach-level monitoring and more rigorous tracking of non-prescribed training exposure would strengthen causal inference and generalizability.

Second, while shooting efficiency and assist-to-turnover ratio are representative indicators, they capture only selected dimensions of tactical execution (Kwon, 2024). More fine-grained performance analytics—such as decision accuracy, defensive coordination timing, shot-selection quality, off-ball movement efficiency, opponent-pressure context, or passing-network efficiency—are recommended to track the dynamic reorganization of perceptual–action coupling during training adaptation.

Third, psychological variables were derived from self-reported perceived fatigue and enjoyment measures. Although ecologically valid, such indicators remain susceptible to motivational bias, recall bias, and social desirability effects (Orth et al., 2025). Incorporating implicit psychological markers, such as HRV-based stress reactivity, attentional tracking, cognitive-load monitoring, or behavioral indicators of engagement during representative tasks, may enrich mechanistic explanations of psychological mediation.

Fourth, although the mediation model was statistically supported, the quasi-experimental design and Pre–Post assessment structure limit causal interpretation. The temporal ordering among external-load adaptation, psychological readiness, and performance improvement was theoretically specified but not verified at the session-by-session level. Therefore, the observed indirect pathway should be interpreted as mechanistic evidence consistent with the proposed load–psychological readiness–performance framework, rather than as definitive proof of a causal mediation process. Future studies should incorporate repeated session-level or micro-longitudinal designs to clarify temporal precedence among training load, affective regulation, and performance transfer (Suppiah et al., 2021).

Fifth, the sample included only male sub-elite collegiate basketball athletes. Adaptation pathways may differ across sex, developmental stages, and competitive levels (Qiu et al., 2025). Therefore, the findings may not generalize directly to female athletes, youth players, recreational participants, or professional teams, where maturity status, tactical expertise, competitive pressure, training history, and coaching ecology may differ substantially. The proposed mechanistic model should therefore be validated in youth training programs, women’s basketball, and professional competition environments.

Finally, the intervention spanned an 8-week off-season period. It remains unclear whether the observed psychological readiness–performance relationship can withstand the cumulative demands of an in-season schedule, including travel fatigue, opponent variability, competitive stress, and heightened psychological pressure (Dudley et al., 2023). Future longitudinal research conducted during active competition will be essential to verify these adaptations’ stability and ecological validity.

Despite these limitations, this study establishes a mechanistic framework integrating physical load, psychological readiness, and decision efficiency, providing a robust foundation for precision constraint design. Future research could extend these findings by exploring real-time constraint regulation strategies, AI-assisted tactical recognition systems, session-level psychological monitoring, and multi-modal physiological–psychological–biomechanical profiling to optimize individualized adaptive efficiency in team-sport training.

## 5 Conclusion

This study confirmed that a constraints-led, game-based basketball training approach improves competitive performance by enhancing the interaction between physical engagement and psychological readiness. The three prespecified hypotheses were supported. First, the constraints-led group showed greater gains in external-load engagement and technical–tactical performance than the technique-then-scrimmage control group. Second, psychological readiness served as a significant indirect pathway linking external-load adaptation to performance improvement. Third, this mediated pathway was stronger among lower-skill athletes, indicating that affective and motivational regulation may be especially important when perceptual–motor patterns are still developing.

Despite equivalent internal-load exposure, only the constraints-led group showed clear transfer to shooting efficiency and decision-making effectiveness. These findings indicate that performance enhancement arises not from increased workload alone, but from the more efficient organization of perception–action regulation under realistic task demands. Reduced fatigue and increased enjoyment appear to support this adaptive process by helping athletes remain engaged, responsive, and tactically effective within representative game situations.

Practically, these results support shifting training priorities from volume accumulation to engagement quality. Coaches may benefit from designing practice tasks that preserve game-relevant information, challenge decision-making, and monitor psychological readiness alongside physical load. Simple indicators such as perceived fatigue and enjoyment may help determine whether athletes are operating within an effective learning environment.

In summary, constraints-led, game-based training enhances basketball performance by improving how athletes interact with game information and by strengthening the psychological readiness required for adaptive execution. Future research should verify the durability of these mechanisms during competition periods and incorporate more granular behavioral, tactical, and physiological indicators to refine individualized training prescriptions.

## Data Availability

The original contributions presented in the study are included in the article/Supplementary material, further inquiries can be directed to the corresponding authors. Additional supporting information is available in the Supplementary material.
