# Supplementary material for: Psychological readiness mediates performance transfer in constraints-led, game-based basketball training: evidence from a quasi-experimental study
Source: Front Psychol. 2026 Jun 11;17:1735248. doi: 10.3389/fpsyg.2026.1735248 (PMC13293939; doi:10.3389/fpsyg.2026.1735248)
Supplement: Supplementary file 1 [file Table_1.docx]

**S-Table 1. Variable Definitions, Measurement Scales, and Coding Scheme**

| **Variable Category** | **Variable** | **Scale / Unit** | **Coding / Directionality** | **Description** |
| --- | --- | --- | --- | --- |
| **External Load** | Running Distance | meters | Higher = more workload | Total distance covered during session |
|  | PlayerLoad™ | AU | Higher = more workload | Composite mechanical demand; Catapult device |
|  | Accelerations | count | Higher = more explosive actions | Total high-intensity accelerations |
|  | Jumps | count | Higher = more plyometric actions | Total vertical efforts |
| **Internal Load** | Avg HR | bpm | Higher = more exertion | Mean heart rate during training |
|  | Session-RPE | 1–10 | Higher = more exertion | Borg CR10 perceived effort scale |
|  | Fatigue | 1–5 Likert | Higher = more fatigue | Self-reported fatigue |
| **Psychological** | Self-Efficacy (ESE) | 12–36 | Higher = better | Sport confidence measure |
|  | Anxiety | 8–28 | Higher = worse | Competitive anxiety scale |
|  | Flow State | 16–48 | Higher = better | State absorption and control |
|  | Cohesion (COH) | 12–40 | Higher = stronger | Group integration and bonding |
| **Performance** | FG% | % | Higher = better | Field goal shooting efficiency |
|  | AST/TO | Ratio | Higher = better | Assist-to-turnover tactical metric |

**Note.** All outcomes were transformed into Δ scores (Post – Pre). For internal load and anxiety, reductions were recoded so that larger Δ = more favorable adaptation.

**S-Table 2. Descriptive Statistics for Change Scores (Δ)**

| **Variable** | **ΔEXP Mean ± SD** | **95% CI** | **ΔCTRL Mean ± SD** | **95% CI** | ***p* (Welch)** | **Hedges *g*** |
| --- | --- | --- | --- | --- | --- | --- |
| Running Distance (m) | +597 ± 150 | [536, 658] | +240 ± 160 | [173, 307] | <0.001*** | 0.88 |
| PlayerLoad | +18.3 ± 6.9 | [15.3, 21.3] | +7.4 ± 7.8 | [4.1, 10.7] | <0.001*** | 0.77 |
| Accelerations | +3.1 ± 2.6 | [2.1, 4.1] | +0.8 ± 2.9 | [–0.3, 1.9] | 0.005** | 0.63 |
| Jumps | +4.4 ± 3.8 | [3.0, 5.8] | +1.1 ± 4.1 | [–0.4, 2.6] | 0.012* | 0.55 |
| Self-Efficacy | +6.6 ± 3.9 | [4.9, 8.3] | +1.4 ± 4.1 | [–0.3, 3.1] | 0.001** | 0.74 |
| Anxiety | –2.0 ± 2.6 | [–3.1, –0.9] | –0.4 ± 2.8 | [–1.5, 0.7] | 0.008** | 0.59 |
| Flow State | +3.7 ± 3.0 | [2.5, 4.9] | +0.6 ± 3.3 | [–0.7, 1.9] | 0.007** | 0.62 |
| Team Cohesion | +5.8 ± 4.9 | [3.8, 7.8] | +1.1 ± 5.2 | [–1.0, 3.2] | 0.003** | 0.69 |
| FG% | +3.3 ± 1.1 | [2.8, 3.8] | +0.2 ± 1.3 | [–0.3, 0.7] | 0.001** | 0.75 |
| AST/TO Ratio | +0.18 ± 0.07 | [0.14, 0.22] | 0.00 ± 0.08 | [–0.03, 0.03] | <0.001*** | 0.82 |

**Note.** Same statistical analysis as Table 2 but presented in extended confidence format for transparency.

**S-Table 3. Full SEM Path Matrix: Standardized Coefficients**

| **Path** | **Estimate (β)** | **SE** | **95% CI** | ***p*** |
| --- | --- | --- | --- | --- |
| ΔExternal Load → ΔPsych Index | 0.52 | 0.10 | [0.33, 0.70] | <0.001*** |
| ΔPsych Index → ΔPerformance | 0.61 | 0.08 | [0.44, 0.77] | <0.001*** |
| ΔExternal Load → ΔPerformance (direct) | 0.21 | 0.11 | [−0.02, 0.43] | 0.072 |
| ΔInternal Load → ΔPsych Index | 0.41 | 0.09 | [0.22, 0.57] | 0.002** |
| ΔRecovery → ΔPsych Index | 0.38 | 0.10 | [0.14, 0.59] | 0.003** |
| ΔExternal Load → ΔInternal Load | 0.47 | 0.07 | [0.30, 0.62] | <0.001*** |

**Note.** Supplementary to main model paths to enhance replicability.

**S-Table 4. Constraint and Invariance Indices for Moderation SEM**

| **Constraint tested** | **χ²** | **df** | **χ²/df** | **Δχ² (*p*)** | **CFI** | **ΔCFI** | **RMSEA** | **Interpretation** |
| --- | --- | --- | --- | --- | --- | --- | --- | --- |
| Unconstrained | 18.32 | 13 | 1.36 | — | 0.961 | — | 0.043 | Excellent baseline fit |
| Constrained (paths equal) | 27.06 | 17 | 1.57 | 8.74 (0.013)* | 0.934 | 0.027 | 0.049 | **Path differences significant** |

**Note.** ΔCFI > 0.010 and Δχ² *p* < 0.05 indicate significant between-group differences (Cheung & Rensvold, 2002). Skill-level moderation confirmed.
